# Nanotoxicity Overview: Nano-Threat to Susceptible Populations

**DOI:** 10.3390/ijms15033671

**Published:** 2014-02-28

**Authors:** Yang Li, Yi Zhang, Bing Yan

**Affiliations:** School of Chemistry and Chemical Engineering, Shandong University, Jinan 250100, China; E-Mails: yangsdu@163.com (Y.L.); zhangyi0622@gmail.com (Y.Z.)

**Keywords:** nanotoxicity, susceptible populations, health risk, aged population, disease

## Abstract

Due to the increasing applications of nanomaterials and nanotechnology, potential danger of nanoparticle exposure has become a critical issue. However, recent nanotoxicity studies have mainly focused on the health risks to healthy adult population. The nanotoxicity effects on susceptible populations (such as pregnant, neonate, diseased, and aged populations) have been overlooked. Due to the alterations in physiological structures and functions in susceptible populations, they often suffer more damage from the same exposure. Thus, it is urgent to understand the effects of nanoparticle exposure on these populations. In order to fill this gap, the potential effects of nanoparticles to pregnant females, neonate, diseased, and aged population, as well as the possible underlying mechanisms are reviewed in this article. Investigations show that responses from susceptible population to nanoparticle exposure are often more severe. Reduced protection mechanism, compromised immunity, and impaired self-repair ability in these susceptible populations may contribute to the aggravated toxicity effects. This review will help minimize adverse effects of nanoparticles to susceptible population in future nanotechnology applications.

## 1. Introduction

Nanotechnology has been advancing rapidly in many fields. It has been applied in various industrial sectors and utilized in more than 1300 marketed consumer products. In biomedicine, nanoparticles provide unprecedented advantages as multifunctional drug delivery carriers, for controlled release, and as biological probes. Nanotechnology may change the current state of medicine in several ways. First, it may provide highly selective and targeted therapeutics, thereby dramatically increasing the efficacy and decreasing the side effects of current therapeutics. Second, it may revolutionize diagnostic and prognostic evaluations by increasing efficiency. Third, drug development may be significantly impacted by nanotechnology.

Despite the numerous benefits of nanotechnology applications, the potential dangers from nanoparticle exposure cannot be ignored. Nanoparticles may damage organisms. *In vitro*, they break DNA helices, disrupt gene expression, and lead to mitochondrial perturbation through an oxidative stress-related mechanism [1–3]. *In vivo*, they induce inflammation and stimulate or suppress the immune system [4–6]. However, a good understanding of nanotoxicity has yet to be achieved. Furthermore, recent nanotoxicity studies have primarily focused on the responses of adult healthy animals, the representative models of healthy adult humans; therefore the effects of nanoparticles on susceptible populations are not well known.

There are many reasons why understanding the effects of nanoparticles in susceptible populations is necessary. Due to the alterations (in most cases, deterioration) in physiological structures and functions in susceptible populations, nanoparticles may exhibit unusual adsorption, distribution, metabolism, and excretion profiles. The impaired or immature protective/repair functions of these populations may lead to aggravated toxic consequences compared with healthy populations. Furthermore, the induction of oxidative stress and inflammation is the major mechanism of nanotoxicity [7]. Weaker antioxidative and immune functions in certain susceptible populations may magnify nanotoxicity.

Available nanotoxicity reviews primarily focus on the potential adverse effects of nanoparticles on healthy adults after unintentional or intentional administration. In this article, we summarize the current knowledge of the effects of nanoparticles on pregnant, neonate, diseased, and aged populations, based on the literature, emphasizing the different responses of susceptible and healthy populations to nanoparticle exposure.

## 2. Nanotoxicity in Pregnant Females and Neonates

In female mammals, the gestation period is accompanied by dramatic alterations in the neuroendocrine network. These alterations are critical to initiating and maintaining the pregnancy, fetal growth and development, and parturition [8,9]. However, these alterations also sensitize pregnant females to exogenous stimuli. One example is the increased sensitivity of many pregnant females to allergens. Approximately one-third of pregnant asthmatics experience worsened symptoms [10].

The susceptibility of pregnant females to nanoparticle exposure is two-fold. First, the neuroendocrine changes during pregnancy may intensify the adverse effects of nanoparticle exposure compared with non-pregnant females. Second, it is widely recognized that nanoparticles can cross the placenta and enter the fetus [11–14] (Table 1). The fetus has no protective mechanisms and is sensitive to toxins, especially during embryonic development [15].

### 2.1. Effects on Health during Pregnancy

Nanoparticle-induced toxicity can be amplified in the pregnant population. A single intranasal administration of titanium dioxide nanoparticles caused no reaction in non-pregnant BALB/c mice. However, the same treatment caused a robust and persistent acute inflammation in pregnant BALB/c mice, as shown by the upregulation of inflammation-associated genes in the lungs [18,19]. This inflammation may be partially caused by the suppression of cell-mediated immunity in pregnant females [20]. The health of the offspring was also affected by this exposure, as indicated by increased susceptibility to asthma [18]. Inhalation and intratracheal instillation of carbon nanoparticles caused pulmonary inflammation in dams and DNA strand breaks in the livers of both dams and offspring [21].

The effects on pregnant animals vary with the chemical nature of the nanoparticles. After repeated oral administrations of multi-wall carbon nanotubes, all pregnant Sprague-Dawley rats survived. Compared with the untreated control group, the maternal body weight, food consumption, and oxidant-antioxidant balance in the liver did not change, although there was a decrease in thymus weight after exposure to a high dose [22]. In another study, an oral dose of platinum nanoparticles did not impact the health of ICR mice, as indicated by normal blood biochemical parameters [23]. Due to the limited number of studies, we are far from understanding the relationship between nanoparticle properties and their potential effects on pregnancy. More studies are required in this area. Considering that fetal development depends exclusively on maternal physiological health, monitoring the nanoparticle-induced health risk to fetal development is urgently required.

### 2.2. Effects on Fetal Development

Recent studies have found that nanoparticle exposure decreased the gestational success rate. Intravenous injection of silica (70 nm) and titanium dioxide nanoparticles (35 nm) in pregnant BALB/c mice significantly increased fetal resorption rates. Nanoparticles also induced structural and functional abnormalities in the placenta, eventually leading to placental dysfunction. Possible mechanisms include the activation of a coagulation pathway and the induction of oxidative stress after nanoparticle exposure [24]. A single oral administration of functionalized carbon nanotubes (10 mg/kg) to pregnant CD-1 mice increased the rate of fetal resorption [25]. In another study, inhaled cadmium oxide nanoparticles decreased the placental weight in pregnant CD-1 mice. Higher doses also decreased the incidence of pregnancy. Prevention of implantation or nanoparticle-induced death of implanted blastocysts were suggested as possible mechanisms [26].

Organs, such as the yolk sac and placenta, provide nutrients and hormones critical for fetal development. Nanoparticles may enter these tissues. For example, platinum nanoparticles and PEG-coated quantum dots translocated to the uterus and placenta of pregnant rodents after intraperitoneal exposure; however, accumulation in the fetus was not detected [13,23]. After intravenous injection of silver nanoparticles (50 nm) in pregnant CD-1 mice, nanoparticles were found in extra-embryonic tissues including the visceral yolk sac and endometrium, but not in the embryo [27]. Adverse outcomes of such intrusions have been reported. *In vivo* studies in rodents indicated that exposure to nanoparticles led to miscarriage, fetal malformations [28], and retarded neonatal growth rate [23,26]. Nanoparticles might cause toxicity to the embryo by destroying the redox equilibrium in the placenta [28], inducing apoptosis in blastocysts [29,30], and inhibiting the differentiation of embryonic stem cells [31].

Nanoparticles may enter the embryo and fetus and cause toxicity (Table 2). Nanoparticles in maternal circulation use different pathways to enter the offspring, including lactation [26,32,33]. *In vivo* studies showed that uncoated CdTe/CdS core/shell quantum dots (<4 nm) [11], polystyrene nanobeads (<240 nm) [12], and silver nanoparticles [27] were distributed in embryos or the fetus after administration to the dam. After intravenous injection, silica (70 nm) and titanium dioxide nanoparticles (35 nm) were found in the placenta, fetal liver, and fetal brain [24]. In one study, the parietal endoderm was separated from gestational mice at day 7.5 and exposed to polystyrene nanoparticles for 12 h. Small carboxylic (20 nm) and larger (200 nm) amine-modified polystyrene nanoparticles were found in the embryo, leading to the inhibition of embryonic growth [17]. *Ex vivo* studies showed that the surface, charge, and size of nanoparticles determined their capacity for placental penetration [12,16,17] (Table 2).

Although it is protected by the placental barrier, the fetus is particularly vulnerable. The leakage of nanoparticles across the placenta exposes important organs to nanoparticles and may induce oxidative stress and inflammation in the fetus. Moreover, maternal inflammatory cytokines induced by nanoparticles can also cross the placenta and affect fetal brain development [36,37]. After maternal exposure to nanoparticles, toxicity to the nervous system and reproductive system of neonates has been reported, and these toxic effects are summarized in Tables 3 and 4.

**Figure 1.**
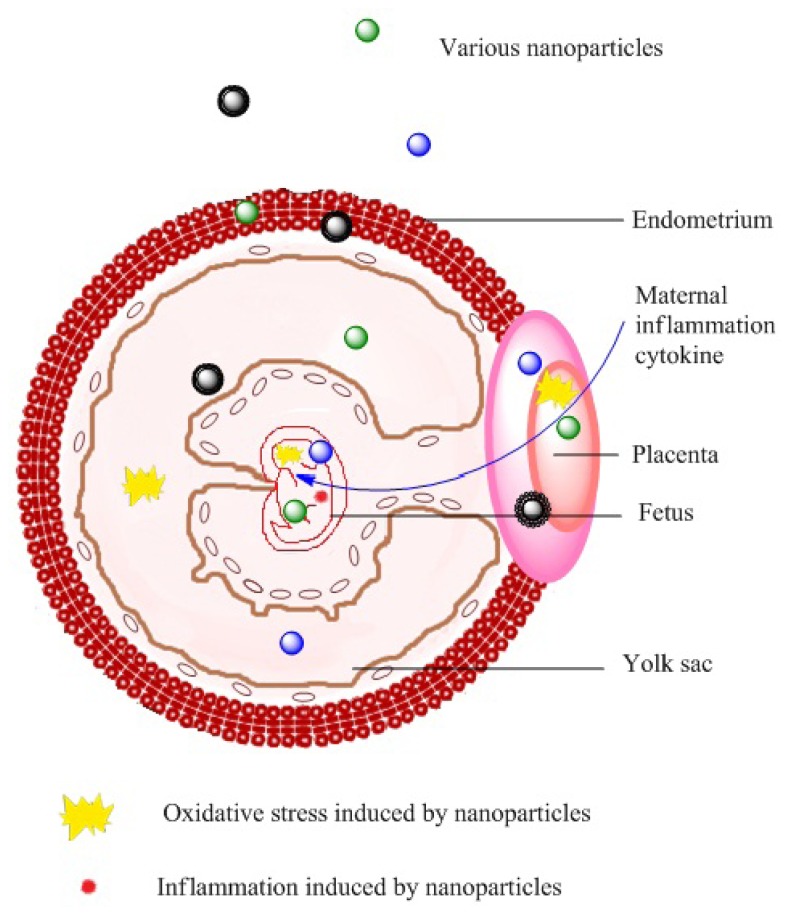
Neonatal toxicity as a result of nanoparticle exposure to pregnant females. Nanoparticles in circulation enter the placenta, endometrium, yolk sac, or fetus, inducing oxidative stress and inflammation. These perturbations lead to the placental dysfunction, retarded neonatal growth, fetal malformations, and neurotoxicity or reproductive toxicity in offspring. Maternal inflammatory cytokines induced by nanoparticles also enter the fetus and affect fetal brain development.

Maternal exposure to nanoparticles may also affect the health of offspring through other mechanisms. For example, exposure of pregnant ICR mice to carbon black nanoparticles induced renal abnormalities similar to tubulointerstitial fibrosis in the kidneys of the offspring [49]. As a secondary consequence of the immigration of cytokines from dams to fetuses after maternal exposure to nanoparticles, alterations in gene expression and DNA damage in the liver of the offspring were observed [47,50]. Mechanistically, these changes might be due to the high rate of cell division in the fetus and the immature repair capability for DNA damage. These damages may increase the susceptibility of the offspring to cancer and other diseases [51]. The above findings are summarized in Figure 1.

## 3. Nanoparticle Toxicity in Diseased Populations

The inherent physicochemical properties of nanoparticles and the responses of biological systems to nanoparticle exposure together determine nanoparticle biocompatibility. The physiological responses to nanoparticles primarily include accessibility to circulating nanoparticles (distribution) and the capacity to degrade (metabolism) and excrete them from the body (excretion). Some physiological structures, such as the blood-brain barrier, protect neuronal systems from nanoparticle toxicity by blocking their entry. The liver is the organ primarily exposed. This organ of the reticuloendothelial system takes up most nanoparticles in circulation; some will be degraded and ultimately excreted [52,53]. In healthy individuals, these physiological functions effectively protect the body and minimize the toxicity caused by nanoparticle exposure. However, in diseased populations, some of these physiological functionalities are disabled, resulting in the risk of toxicity after nanoparticle exposure. In this section, we will summarize the effects of nanoparticles on populations with cardiovascular disease, chronic respiratory disease, and hepatitis.

### 3.1. The Effects of Nanoparticles on Subjects with Cardiovascular Diseases

Cardiovascular disease refers to chronic disorders of the heart and blood vessels, and it is a leading cause of death worldwide. According to the WHO (2005), approximately 20 million people will die of cardiovascular diseases in 2015, accounting for 30% of all deaths worldwide [54]. Particulate matter in the environment has recently been found to be an important trigger for cardiovascular diseases [55,56]. This finding raises the concern that inhaled nanoparticles or those administered by other routes may cause or aggravate cardiovascular diseases. Administration of nanoparticles to healthy animals through various routes significantly impaired vasodilator responses in coronary arterioles [57,58] and mesenteric microvasculature function [58]. *In vitro*, iron oxide nanoparticles induced cytoplasmic vacuolation, mitochondrial swelling, and cell death in human aortic endothelial cells. Moreover, iron oxide nanoparticles stimulated nitric oxide production and the adhesion of monocytes, both of which are early steps of atherosclerosis [59]. These results indicate that nanoparticle exposure not only poses a risk for the acquisition of cardiovascular disease in healthy people but may also threaten individuals who already suffer from cardiovascular diseases.

Studies using specific animal models provide valuable insight into the effects of nanoparticles in the population with cardiovascular defects. Atherosclerosis is involved in almost all cardiovascular diseases [60–62]; animal models of atherosclerosis provide a useful tool to study various cardiovascular diseases. Apolipoprotein E (apoE) is a glycoprotein that forms a constituent of lipoproteins and helps to clear chylomicrons and very low–density lipoprotein (VLDL) remnants in blood [63]. Deletion of the apoE gene in animals (apoE-deficient, apoE^−/−^, animals) causes problems, including atherosclerosis [64,65]. Using this model, the effects of nanoparticle exposure on cardiovascular populations have been investigated. Long-term (five months) exposure of apoE^−/−^ mice to nickel nanoparticles via inhalation accelerated the progression of atherosclerosis in the ascending aorta and enhanced the expression of related genes [66]. A single intrapharyngeal instillation of SWCNTs to apoE^−/−^ mice caused mtDNA damage and changes in aortic mitochondrial glutathione and protein carbonyl levels, all indicators of cardiovascular damage. Long-term exposure (eight weeks) also accelerated plaque formation in both the aorta and the brachiocephalic artery [67]. Another atherosclerosis model was established by feeding Sprague-Dawley rats vitamin D3 and high-lipid chow [68]. Intravenous injection of MWCNTs in these animals led to aggravated atherosclerosis characterized by increased aorta injury and calcification. Disrupted endothelial tight junctions upon exposure to MWCNTs indicated that turbulence in endothelial function may trigger atherosclerosis [69]. Pulmonary inflammation animal models were used to clarify nanoparticles’ effects on cardiovascular functions. Nanoparticles, such as carbon black, carbon nanotubes, or TiO_2_ nanoparticles impaired coagulatory functions of lipopolysaccharide-treated ICR mice and this disturbance depended on particle size. Mechanistic investigation indicated that the exacerbated permeability in pulmonary vessel may be one of the reasons [70–72].

**Figure 2.**
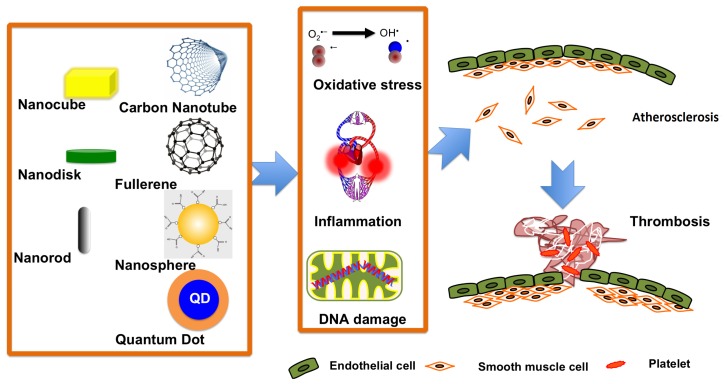
A schematic showing the methods through which nanoparticles aggravate cardiovascular conditions in diseased populations. Varieties of nanoparticles in the circulatory system induce oxidative stress, inflammation, or aortic mitochondrial DNA damage. These effects consequently accelerate atherosclerotic lesions, ultimately leading to thrombosis. In this figure, the migration of smooth muscle cells to the intima is simplified by combining the initial and progression steps of atherosclerosis. Thrombosis can lead to the obstruction of blood flow and, thus, have lethal consequences.

Many factors such as oxidative stress [73], inflammation [74], mitochondrial DNA damage [75,76] in the aorta, and damage to vessel endothelial cells [77] have been proposed to trigger atherosclerosis. Studies have suggested that nanoparticle exposure aggravates all of these potentially triggering factors [59,67,78–80] and consequently accelerates atherosclerosis progression through platelet aggregation and vascular thrombosis [81,82] (Figure 2). Although nanoparticles hold promise as targeted drug delivery carriers to treat cardiovascular diseases [83,84], special attention should be paid to their potential adverse effects.

### 3.2. Effects of Nanoparticles on Populations with Chronic Respiratory Disease

Chronic respiratory diseases, especially asthma and chronic obstructive pulmonary diseases (COPD) such as chronic bronchitis, are common in countries with aging populations, a high smoking rate, and increased environmental pollution [85]. In 2004, asthma affected 300 million people globally [86]. COPD affected 329 million people and led to the death of over three million people in 2011 [87]. Both, asthma and COPD, involve inflammation of the airways and the consequent narrowing of airway tubes because of the thickening of smooth muscle or increased mucus in the airway tube. The airways of patients suffering from chronic respiratory diseases are sensitive to irritants such as airborne particles. These allergens stimulate basophils and mast cells and produce persistent inflammation in the airways, resulting in deteriorating conditions [88–90].

The use of nanoparticles in pulmonary imaging, diagnosis, and treatment of respiratory diseases has been promising [91]. However, recent research has suggested that nanoparticle exposure may exacerbate respiratory diseases. Particulate matter shows increased deposition in the lungs of asthma patients compared with healthy subjects. Early studies have shown that ultrafine particle deposition was increased in asthmatics [92]. A study in humans confirmed that inhaled carbon nanoparticles (23 nm) were more easily deposited in the lungs of asthmatic patients compared to healthy subjects, as indicated by 74% higher particle numbers after inhalation while resting and 43% higher during exercise [93]. As a result of airway obstruction, asthmatics display increased lung residual volume, which is predicted to account for the enhanced diffusion deposition. The higher respiratory dose received by asthma sufferers compared with healthy subjects who receive the same exposure may be an important reason for the higher susceptibility of asthma subjects to nanoparticle exposure.

Moreover, nanoparticles deposited in airways persistently trigger inflammation, which may synergistically aggravate pre-existing conditions[94]. Both, clinical data and laboratory research, have shown that inhaled particulate matter worsen asthma symptoms. For example, diesel exhaust particles were found to stimulate and aggravate asthma in animal models [95,96]. The initiation of inflammation in the airways and the lungs [97–100] and damage to airway epithelial cells [101,102] after nanoparticle exposure have been reported. MWCNTs stimulate the metaplasia of goblet cells and increased mucin secretion in airway tubes [103]. In the alveoli, the process of nanoparticle phagocytosis by macrophages led to chemotaxis, the complement system cascade, and inflammatory cell response. Attacks on respiratory cells caused by inflammation require an extended time to clear [104].

To study the effects of nanoparticle exposure on respiratory disease subjects, respiratory disease animal models have been generated by treatment with endotoxin (usually lipopolysaccharide; LPS), chemicals, or ovalbumin (OVA; Table 5).

#### 3.2.1. LPS-Induced Animal Asthma Model

LPS administration to rodents can partially simulate asthma conditions in animals [117]. In one study, Sprague-Dawley rats were nasally aspirated with LPS one day before the intratracheal instillation of MWCNTs and carbon black. Twenty-four days after exposure to MWCNTs (but not carbon black), obvious lung injury and the formation of pulmonary fibrosis were observed in LPS-exposed rats [115]. These results showed that MWCNT exposure might lead to a fibrogenic response in patients with respiratory disease. In another study, MWCNTs and SWCNTs were intratracheally injected into ICR mice simultaneously with LPS. After 24 h of exposure, both CNTs enhanced LPS-stimulated expression of inflammatory cytokines[tumor necrosis factor-α (TNF-α) and interleukin-1β (IL-1β)] and chemokines[macrophage inflammatory protein-1α (MIP-1α), monocyte chemotactic protein-1 (MCP-1), and keratinocyte-derived chemoattractant (KC)] in lung tissues and blood [71]. The synergistic effects of nanoparticles with LPS are likely size dependent, given that SWCNTs show a stronger effect than MWCNTs. In a similar investigation, 14-nm carbon black nanoparticles significantly aggravated lung inflammation and pulmonary edema, whereas larger nanoparticles (56 nm) did not [70]. In another study, small latex nanoparticles (less than 50 nm) amplified the lung inflammation elicited by LPS in ICR mice compared with large nanoparticles [109].

#### 3.2.2. OVA-Induced Animal Asthma Model

In contrast to LPS, which stimulates inflammation in the respiratory system mediated by proinflammatory cytokines and chemokines, allergens, such as OVA, cause airway inflammation via immunoglobulin production as a consequence of activated adaptive immunity. The adjuvant effects of nanoparticles have been well established [118]. The dysregulation of immune reactivity in almost all respiratory diseases has led to the postulation that nanoparticle exposure exacerbates respiratory conditions through their adjuvant effects.

Nanoparticles may interfere with different molecular signaling pathways that mediate respiratory allergies. Diesel exhaust particles promote the local and systemic dysregulation of Th immunity in mice by enhancing both antigen-presenting cell and OVA-specific Th responses [106]. After concomitant intratracheal instillation of OVA and carbon nanoparticles in mice, IL-5 expression, activation of Th2-like lymphocytes, and eosinophilic inflammation were observed [108]. An enhancement of neutrophilic rather than eosinophilic lung inflammation by latex nanoparticles in OVA-sensitized mice has been reported [109]. Simultaneous intranasal exposure to OVA and TiO_2_ nanoparticles (14 and 29 nm) to mice amplifies the effects of OVA in inducing cell immunity as manifested by the increased number of lung-draining peribronchial lymph node cells and the production of OVA-specific Th2 cytokines (interleukin (IL)-4 (IL-4), IL-5, IL-10, and IL-13) [110]. Moreover, TiO_2_ nanoparticles [110] and CNTs [103,112] augment animal humoral immunity, as shown by an increased serum level of OVA-specific immunoglobulin E (IgE) and immunoglobulin IgG1 (IgG1). The capability to augment humoral immunity is likely shape-dependent because spherical carbon nanoparticles only stimulated moderate production of OVA-specific IgE [112]. In another study, mice were intranasally instilled with ultrafine particles (less than 150 nm) from diesel exhaust particles (DEP) one day before being challenged with OVA, and an enhanced Th2 polarization and allergic inflammatory response in both upper and lower airways were observed [119]. A mechanistic investigation indicated that the adjuvant effects might be related to the oxidant potential of nanoparticles.

In the respiratory system, nanoparticles induce airway fibrosis [120,121]. Transforming growth factor-β1 (TGF-β1) and platelet-derived growth factor-AA (PDGF-AA), a potent fibroblast mitogen that is important for expanding fibroblasts, are both needed for a fibrogenic response. Experiments have shown that pre-existing inflammation in the airway was indispensable for the fibrosis induced by exposure to nanomaterials. MWCNT exposure alone only induced PDGF-AA, while administration of OVA alone induced TGF-β1. Neither causes fibrosis in the animals’ airway alone. After concomitant exposure to both MWCNTs and OVA in mice, the expression of growth factors and cytokines, such as IL-5, was activated, leading to fibrosis in the bronchioles [111]. This finding revealed another process through which nanoparticle exposure can cause injury in asthmatic populations.

#### 3.2.3. Chemically Induced Animal Asthma Model

Isocyanates irritate the human respiratory system, leading to asthma [122]. This property has been used to establish asthmatic animal models [123]. In a toluene diisocyanate-induced mouse model of asthma, administration of TiO_2_ or Au nanoparticles increased pulmonary inflammation and airway hyperreactivity [116].

**Figure 3.**
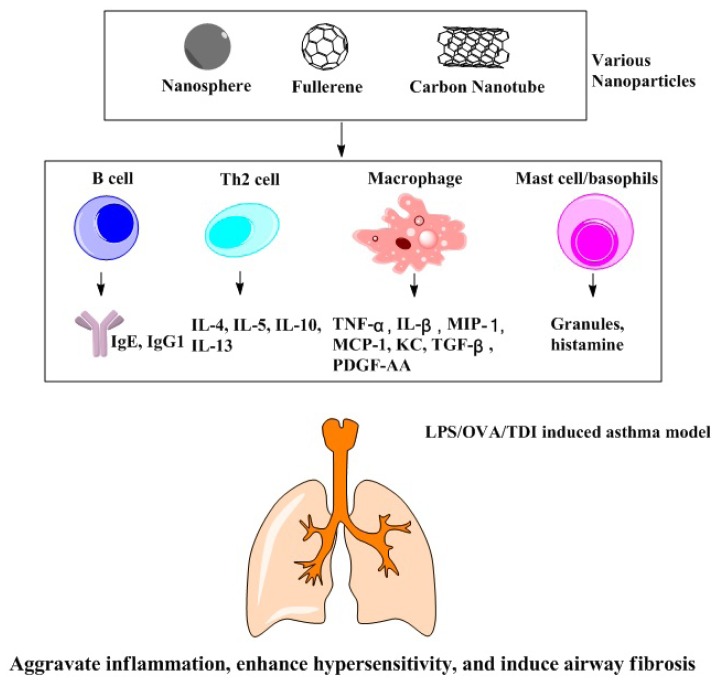
Nanoparticle-induced aggravation of respiratory symptoms in animal models of asthma. Nanoparticles aggravate asthma symptoms in the following three ways: Nanoparticles stimulate humoral immunity (the production of immunoglobulins IgE and IgG1) and the expression of inflammatory cytokines and chemokines, leading to the aggravation of inflammation in the respiratory tubes; Nanoparticles stimulate the expression of PDGF-AA and TGF-β1, leading to airway fibrosis; Nanoparticles also activate Th2 cells, leading to the enhancement of hypersensitivity.

The above studies suggest that nanoparticle exposure may aggravate respiratory conditions in animals through two major mechanisms. First, nanoparticles amplify pre-existing inflammation in the respiratory tubes by enhancing the levels of inflammatory factors or humoral immunity. Second, they stimulate and enhance hypersensitivity, which is primarily mediated by Th2 cells (Figure 3). Understanding these mechanisms helps design measures to minimize the adverse effects of nanoparticles in this population.

### 3.3. Effects of Nanoparticles on Hepatitis Patients

As a major detoxification organ, the liver is susceptible to injury from multiple risk factors. Hepatitis is the most common liver disease and is characterized by injury to or dysfunction of hepatocytes. Immune injury caused by activated macrophages and NK cells in the liver is an important underlying cause of hepatitis [124]. Based on its etiology, hepatitis can be divided into viral hepatitis, non-alcoholic steatohepatitis, alcoholic hepatitis, and drug-induced hepatitis. These diseases are caused by viral infection, metabolic disorders, and alcohol and drug metabolites. In Asia and eastern Europe, viral hepatitis affects 70%–90% of the population [125]. In Western countries, 20%–30% of the general population is affected by non-alcoholic steatohepatitis [126]. Hepatitis has become a serious global health problem.

Nanoparticles entering circulation primarily accumulate in the liver because it is a reticuloendothelial system organ. Depending on their chemical nature, nanoparticles are cleared from the liver at different rates. In the liver, nanomaterials are primarily taken up by macrophages, where they activate an inflammatory response through an oxidative stress-mediated mechanism [127–130]. The similarity in the mechanisms of liver damage from nanoparticles and hepatitis causative agents has raised the question of whether nanoparticle exposure can aggravate liver conditions in populations with hepatitis.

Two investigations using animal hepatitis models have provided important insights. In one study, C57BL/6 mice were treated with concanavalin A for 8 h and carbon tetrachloride for 6 weeks to establish acute and chronic liver injury animal models. The effects of gold nanorods were assayed 48 h after exposure. The results showed that gold nanorods exacerbated liver damage in animals with acute liver injury by activating hepatic macrophages, as indicated by the increased area of necrotic hepatocytes, the infiltration of mononuclear cells, and higher serum alanine aminotransferase (ALT) levels. In comparison, gold nanorods did not affect liver fibrosis in chronic hepatic injury models [131]. As concanavalin A and carbon tetrachloride caused liver damage through completely different mechanisms, these findings suggest that gold nanorods intensified the hepatocytic immune injuries caused by concanavalin A, while they did not affect the direct damage induced by carbon tetrachloride.

In another study, a nonalcoholic steatohepatitis model was established by feeding mice a methionine- and choline-deficient (MCD) diet for four weeks. Twenty-four hours and seven days after tail-vein injection of PEG-coated gold nanoparticles (5 mg kg^−1^), a higher level of liver damage was observed through elevated serum ALT and AST levels compared with MCD diet-fed mice. In comparison, these nanoparticles had no effects on healthy control mice. A mechanistic investigation showed that gold nanoparticles stimulate an inflammatory response and accelerated stress-induced apoptosis in the liver [132]. Viral hepatitis and drug-induced hepatitis mainly cause hepatic damage through an immune system-mediated mechanism [133–135]. The above findings suggest that the populations with hepatitis should be particularly aware of nanoparticle exposure [132].

In addition to the above vulnerable populations, the incidence of other disorders such as diabetes mellitus, malnutrition, and mental diseases are increasing [136–139]. Future studies should also focus on the effects of nanoparticle exposure on a wide range of vulnerable populations.

## 4. Nanoparticle Toxicity in the Elderly Population

As the percentage of the aging population grows, humans enter the era of so-called global aging [85]. Aging is a complex physiological process characterized by the decline of cellular and organic functions, including but not limited to impaired protein degradation, regenerative capability, and immune function. The changes in physiological functions make the elderly more prone to certain diseases such as metabolic syndrome, cardiovascular disease, neurological diseases, and cancer.

The potential for environmental or biomedical nanoparticle exposure makes it critical to understand the effects of nanoparticles on the elderly. Clinical data have revealed that inhalation of particulate matter in the air leads to cardiac and pulmonary dysfunctions in the elderly [140–142]. Some recent studies using aged animal models showed that compared with young and adult animals, aged animals are more susceptible to the adverse effects of nanoparticles. For example, when 20-month-old rats (equivalent to 60–80 years old in humans) were administered SiO_2_ nanoparticles (24.1 mg/m^3^; 40 min/day) by inhalation for four weeks, cardiovascular dysfunction was induced, as indicated by myocardial ischemic damage, atrio-ventricular blockage, and increased fibrinogen concentration and blood viscosity [143]. These symptoms were absent in young and adult animals. Moreover, nanoparticle exposure led to more severe pulmonary inflammation when bronchoalveolar lavage parameters and serum histamine levels were considered, indicating that the respiratory system is susceptible to damage in elderly exposed to nanoparticles [143].

Due to the lack of studies, it is difficult to fully evaluate the effects of nanomaterial exposure on the health of the elderly. One important branch of nanomedicine is the development of therapeutics for the treatment of age-related disorders. For example, nanoparticles have been repeatedly reported to affect the function of neurons and are thus a promising method to treat neurodegenerative diseases such as Alzheimer’s and Parkinson’s diseases in the elderly [144,145]. The elderly population is characterized by decreased tissue regenerative and metabolic capabilities compared with the young [146,147]. These changes indicate that when nanoparticle-based therapeutics are developed, suitable aged animal models should be preferentially considered and special attention should be paid to nanotoxicity.

## 5. Conclusions

In summary, the potential effects of nanoparticles on pregnant females, disease populations, and the elderly population, as well as the possible underlying mechanisms of such effects have been reviewed on the basis of animal model investigations. Exposing pregnant animals to nanoparticles (such as titanium dioxide nanoparticles) amplifies pulmonary inflammation. Such exposure may also affect the health of the fetus and the offspring, leading to abnormal fetal development and malfunctions in offspring including reproductive toxicity and neurotoxicity. Maternal exposure to nanoparticles induces inflammatory cytokines that may enter the fetus and induce alterations in gene expression and cause DNA damage. Most diseases are associated with oxidative stress and inflammation. Nanoparticles may aggravate disease conditions by inducing further oxidative stress and inflammation. In cardiovascular patients, nanoparticle exposure accelerates the progression of atherosclerosis by enhancing oxidative stress, inflammation, mitochondrial DNA damage in aortas, and damage to vessel endothelial cells. In chronic respiratory disease, nanoparticle exposure amplifies pre-existing inflammation in the respiratory tubes and enhances hypersensitivity, primarily through a Th2-related mechanism. Nanoparticle exposure selectively exacerbates acute liver damage in a concanavalin A-induced animal model, but not in the chronic liver damage animal model induced by carbon tetrachloride. Nanoparticles aggravate the symptoms of nonalcoholic steatohepatitis in an animal model by stimulating the inflammatory response and accelerating stress-induced apoptosis in the liver. Finally, nanoparticles more readily induce severe pulmonary inflammation and cardiovascular disease in the aged population, likely because of their reduced physiological functions. Therefore, these susceptible populations should avoid nanoparticle exposure if possible. Further studies on models of these and other susceptible populations are warranted.

## Figures and Tables

**Table 1.** Transfer of nanoparticles to fetuses.

Type	Materials	Animals/cells	Mechanism of exposure	Findings	Ref.
*In vivo*	CdTe/CdS core/shell QDs (1.7, 2.6, 3.2 nm)	Kun Ming mice	Intravenous injection of PBS (pH 7.4)-diluted QDs containing 20, 50, 86, or 125 μg Cd 20–22 days after female mice were housed with male mice	QDs were transferred to the fetuses across the placental barrier, smaller QDs transferred more easily, the number of QDs transferred was dose dependent	[11]
*In vivo*	PEG-coated CdSe/ZnS QDS	Wistar rats	Intraperitoneal injection of 0.8 μmol/L QDS on GD 18	QDs were not detected in fetal tissues	[13]
*In vitro*	Gold nanoparticles coated with PEG (15 and 30 nm)	Human placenta	Open perfusion for 5 min, 7.9 × 10^11^ for 15-nm particles and 7.8 × 10^10^ for 30-nm particles	Detection of high levels of nanoparticles soon after perfusion in maternal outflow, no detection of nanoparticles in fetal outflow	[16]
*In vitro*	Gold nanoparticles coated with PEG (10 and 15 nm)	Human placenta	Recirculating perfusion for 6 h, 9.1 × 10^9^ for 10-nm particles and 2.0 × 10^9^ for 15-nm particles	No transplacental transfer of nanoparticles	[16]
*In vitro*	Polystyrene beads (50.80, 240, 500 nm)	Human placenta	Open perfusion for 20 min at 25 μg/mL	Polystyrene beads with diameters up to 240 nm crossed the placental barrier	[12]
*In vivo*	Silicon nanovectors (519, 834, 1000 nm)	Sprague Dawley rats	Intravenous injection on GD 20 at 1.2 × 10^−9^ g/mouse	Fetal silicon levels were higher only in the 519 nm SNV group	[14]
*In vitro*	Amine-modified polystyrene beads (PS; 200 nm), carboxyl-modified PS (20, 100, 500 nm)	BALB/c mice blastocysts	Micro injection of 0.6 (20 nm carboxyl PS), 0.6 (100 nm carboxyl PS), 1.25 (200 nm amine PS), 8 μL (500 nm carboxyl PS) PS via extraembryonic tissue on GD 7.5	20-nm carboxylic PS and 200-nm amine-modified PS were detected in the embryos, while 100- and 500-nm PS were not	[17]

**Table 2.** Developmental toxicity of nanoparticles.

Type	Materials	Animals/cells	Mechanism of exposure	Findings	Ref.
*In vivo*	Cadmium oxide nanoparticles (11 and 15 nm)	CD-1 mice	Inhalation of 100 μg CdO/m^3^/2 days or 230 μg CdO/m^3^/day on 4.5 days post coitus (dpc) to 16.5 dpc	Fetal length and neonatal growth rate decreased	[26]

*In vivo*	TiO_2_ nanoparticles (20.6 nm)	C57BL/6 mice	Inhalation of 42.4 mg UV-Titan/m^3^ 1 h/day on GD 8–18	F2 female descendants’ ESTR germline mutation rates unchanged	[34]

*In vivo*	p-SWCNTs, o-SWCNTs, uo-SWCNTs	CD-1 mice	Intravenous injection of 10 ng, 100 ng, 300 ng, 3 μg, or 30 μg/mouse on 5.5 dpc	Early miscarriages and fetal malformations	[28]

*In vitro*	Silver nanoparticles (13 nm)	ICR mice blastocysts	Incubation of 25 or 50 μmol/L silver nanoparticles on GD 3	Apoptosis and developmental retardation in blastocysts	[29]

*In vitro*	CdSe-core QDs (3.5 nm)	ICR mice blastocysts and morulas	Incubation at 125, 250, or 500 nmol/L for 24 h	Number of apoptotic cells of blastocysts at 250 and 500 nmol/L increased, development of morulas into blastocysts at 250 and 500 nmol/L was blocked, blastocyst development at 125 nmol/L and higher was retarded	[30]

*In vitro*	Amine-modified polystyrene beads (200 nm), carboxyl-modified PS (20, 100, or 500 nm)	BALB/c mice blastocysts	Micro injection via extraembryonic tissue of 0.6, 0.6, 1.25, or 8 μL PS on GD 7.5	Growth inhibition of embryos was detected; translocation in embryos was associated with surface modification and size	[17]
*In vitro*	Silica nanoparticles (10 or 30 nm)	Mouse embryonic stem cells	Incubation at 1, 3, 10, 30, 100 μg/mL for 24 h or 10 days	Inhibition of differentiation of stem cells was detected below cytotoxic concentrations	[31]

*In vivo*	CdSe/ZnS QDs, CdTe QDs	Wistar rat	Intraperitoneal injection on the 6th, 13th, and 18th days of embryogenesis at 5 mg/kg	QDs did not cause any direct embryotoxic or teratogenic effects	[35]

**Table 3.** Neurotoxicity of nanoparticles to offspring.

Type	Materials	Animals/cells	Methods of exposure	Findings	Ref.
*In vivo*	TiO_2_ nanoparticles (97 nm)	C57BL/6BomTac mice	Inhalation of 42.4 mg UV-Titan/m^3^ 1 h/day on GD 8–18	Moderate neurobehavioral alterations in offspring	[38]
*In vivo*	Anantase TiO_2_ nanopowder (2570 nm)	ICR mice	Subcutaneous injection of 100 μg/mouse/time on GD 6, 9, 12, and 15	Alterations in expression of genes related to brain development, central neural system function, and inflammation in offspring	[39]
*In vivo*	Carbon black nanoparticles (Printex 90; 140 nm)	C57BL/6BomTac mice	Instillation of 11, 54, and 268 μg Printex 90/animal on GD 7, 10, 15, and 18	Altered habituation pattern in the open field test	[40]
*In vivo*	Anantase TiO_2_ nanoparticles (25–70 nm)	ICR mice	Subcutaneous injection of 100 μg/mouse/time on GD 6, 9, 12, and 15	Alterations in the cerebral cortex, olfactory bulb, and some regions related to dopamine systems	[41]
*In vivo*	Anantase TiO_2_ nanoparticles (25–70 nm)	ICR mice	Subcutaneous injection of 100 μg/mouse/time on 3, 7, 10, and 14 dpc	Apoptosis in the olfactory bulb of the brain	[42]
*In vivo*	Anantase TiO_2_ nanoparticles (25–70 nm)	ICR mice	Subcutaneous injection at 0.1 mg/mouse/time on GD 6, 9, 12, 15, and 18	Dopamine levels in the prefrontal cortex and neostriatum increased	[43]
*In vivo*	Anantase TiO_2_ nanoparticles (<25 nm)	Sprague-Dawley rats	Oral administration at 100 mg/kg on prenatal day 2–21 or postnatal day 2–21	Short and long-term synaptic plasticity in the rat hippocampal DG area was impaired	[33]
*In vitro*	Polyethylene nanoparticles (33 nm)	Human embryonic stem cells	Incubation at 360 μg/mL for 48 h	Downstream neuronal precursor genes and a patterning marker gene were reduced in expression	[44]

**Table 4.** Reproductive toxicity of nanoparticles to offspring.

Type	Materials	Animals/cells	Method of exposure	Findings	Ref.
*In vivo*	Carbon black nanoparticles (14 nm)	ICR mice	Instillation at 0.2 mg/mouse on GD 7 and 14	Seminiferous tubule vacuolation, decreased DSP, reduced cellular adhesion of seminiferous epithelia	[45]
*In vivo*	DMSA-coated Fe_3_O_4_ nanoparticles (3–9 nm)	Balb/C mice	Intraperitoneal injection at 50, 100, 200, and 300 mg/kg on GD8	Infant growth decreased, testes development was disrupted	[46]
*In vivo*	Titanium dioxide (UV-Titan) nanoparticles (17 nm)	C57BL/6BomTac	Inhalation of 42 mg UV-Titan/m^3^ on GD 8–18 1 h/day	Changes in gene expression related to the retinoic acid signaling pathway in female offspring	[47]
*In vivo*	UV-Titan (20.6 nm), Printex 90 (14 nm)	C57BL/6J mice	Inhalation and intratracheal instillation of 42 mg/m^3^ UV-Titan or 67 μg/animal Printex 90 on GD 8–18 at 1 h/day (UV-Titan) or on GD 7, 10, 15, and 18 (Printex 90)	UV-Titan reduced sperm counts in the F1 generation, time-to-first F2 litter increased in male offspring	[48]
*In vivo*	Anantase TiO_2_ nanoparticles (25–70 nm)	ICR mice	Subcutaneous injection at 100 μg/mouse/time on 3, 7, 10, and 14 dpc	Daily sperm production reduced	[42]

**Table 5.** Studies of nanoparticle toxicity in asthma models.

Type	Materials	Animal/cell model	Mechanism of exposure	Findings	Ref.
*In vivo*	DEP	OVA-induced asthma ICR mice model	Intratracheal injection of 100 μg DEP once a week for 6 weeks	OVA-specific IgG and IgE production were enhanced; IL-5, IL-4, GM-CSF, and IL-2 expression increased; ovalbumin-induced airway inflammation was aggravated	[105]
*In vivo*	DEP	OVA-induced ICR asthma mice model	Intratracheal injection of 100 μg DEP every 2 weeks for 4 weeks (a total of 3 injections)	DEP promoted local and systemic dysregulation of Th immunity in mice by 1. enhancement of antigen-presenting cell (APC) activity including dendritic cells (DC) and 2. enhancement of extrathoracic antigen-specific Th responses	[106]
*In vivo* and *In vitro*	DEP, carbon black (CB)	*In vivo*: OVA-induced Brown Norway asthma rat model. *In vitro*: bone marrow-derived dendritic cells (BMDC)	*In vivo*: Intratracheal instillation of 5 mg/kg DEP or CB once *In vitro*: Exposed to different concentrations of DEP (1–10 μg/mL) for 24 h	Pulmonary inflammation was enhanced; serum OVA-specific IgG and IgE levels increased significantly; glutathione (GSH) levels in lymphocytes were reduced; IL-4 mRNA levels in lung tissue increased	[107]
*In vivo*	Carbon black NP	OVA-induced ICR asthma mice model	Intratracheal injection of 50 μg DEP once a week for 6 weeks	Accelerated OVA-induced expression of IL-5 and activated Th2-like lymphocytes, which together caused eosinophilic inflammation; smaller CB had more prominent aggravation effects	[108]
*In vivo*	Latex nanoparticles (25, 50, and 100 nm)	OVA-induced ICR asthma mice model	Intratracheal injection of 50 or 100 μg latex nanoparticles every week for 6 weeks	Latex nanoparticles enhanced neutrophilic, but not eosinophilic lung inflammation in a size-dependent manner	[109]
*In vivo*	Titanium dioxide nanoparticles (TiO_2_; 250, 260, 29 and 14 nm)	OVA-induced BALB/cANN Crl asthma mice model	Intranasal droplet application on days 0, 1, and 2 (total 200 μg)	Lung-draining peribronchial lymph node cell numbers increased, and OVA-specific Th2 cytokines (IL-4, IL-5, IL-10, and IL-13) were produced	[110]
*In vivo* and *In vitro*	MWCNTs	*In vivo*: OVA-induced ICR asthma mice model *In vitro*: BMDCs	*In vivo*: Intratracheal injection of 25 or 50 μg MWCNT once a week for 6 weeks *In vitro*: exposure to different concentrations of MWCNT (0.1–1 μg/mL) for 24 h	MWCNTs aggravated allergen-induced airway inflammation, Th cytokine and chemokine levels increased, IgG_1_ and IgE levels increased, syngeneic T-cell proliferation increased, and APCs including DC were activated	[103]
*In vivo*	MWCNTs	OVA-induced C57BL/6 asthma mice model	Inhalation of 100 mg/m^3^ MWCNT for 6 h	PDGF, TGF-β1, and IL-5 mRNA levels were elevated, airway fibrosis was induced	[111]
*In vivo*	MWCNTs, SWCNTs	OVA-induced BALB/cAnN Crl asthma mice model	Injection model: subcutaneous injection of 200 μg (single dose) MWCNT or SWCNT into the mouse footpad Intranasal model: Intranasal administration of 400 μg (133 μg per day for 3 days) MWCNT or SWCNT	Serum OVA-specific IgE levels increased, the number of eosinophils in bronchoalveolar lavage fluid (BALF) increased, Th2-associated cytokines in the mediastinal lymph node (MLN) increased, IgG2a levels, TNF-α levels and neutrophil cell numbers increased only in the MWCNT group	[112]
*In vivo* and *In vitro*	SWCNTs	*In vivo*: OVA-induced ICR asthma mice model *In vitro*: BMDCs	*In vivo*: intratracheal administration of 25 or 50 μg SWCNT once a week for 6 weeks *In vitro*: exposed to various concentrations of SWCNT (0.1–10 μg/mL)	Aggravated allergen-induced airway inflammation with mucus hyperplasia, OVA-specific IgG_1_ and IgE and Th cytokine and chemokine levels increased, oxidative stress level was accentuated, dendritic cells were activated	[113]
*In vivo*	DEP	LPS-induced ICR asthma mice model	Intratracheal instillation of 250 μg DEP once	DEP enhanced neutrophilic lung inflammation by the induction of proinflammatory molecules including p65-containing dimer(s) of NF-κB and Toll-like receptors	[95]
*In vivo*	DEP	LPS-induced ICR asthma mice model	Inhalation of DEP at a concentration of 15, 36, or 169 μg/m^3^ once	DEP exacerbated lung inflammation by production of IL-1β and keratinocyte chemoattractant	[96]
*In vivo*	Washed DEP, organic chemicals of DEP (DEP-OC)	LPS-induced ICR asthma mice model	Intratracheal instillation of 125 μg washed DEP or DEP-OC once	Residual carbonaceous DEP nuclei mainly contribute to the aggravation of LPS-induced lung inflammation	[114]
*In vivo*	MWCNTs, CB nanoparticles	LPS-induced Sprague-Dawley asthma rat model	Intratracheal instillation at 4 mg/kg once	MWCNTs but not CB caused more obvious lung injury and led to the formation of pulmonary fibrosis in rats with pre-existing inflammatory conditions	[115]
*In vivo*	SWCNTs, MWCNTs	LPS-induced ICR asthma mice model	Intratracheal instillation at dose of 4 mg/kg once	Both CNTs enhanced LPS-stimulated expression of inflammatory cytokines and chemokines in lung tissue and in circulation, including IL-1β, MIP-1α, MCP-1, and keratinocyte-derived chemo-attractants; the effects were more prominent with SWCNT than with MWCN	[71]
*In vivo*	CB nanoparticles (14, 56 nm)	LPS-induced ICR asthma mice model	Intratracheal administration at dose of 4 mg/kg once	CB nanoparticles of 14 nm but not 56 nm aggravated lung inflammation and pulmonary edema by inducing the expression of IL-1β, MIP-1α and keratinocyte chemoattractant	[70]
*In vivo*	Latex nanoparticles (25, 50, and 100 nm)	LPS-induced ICR asthma mice model	Intratracheal injection of 50 or 100 μg latex nanoparticles every week for 6 weeks	Latex nanoparticles aggravated lung inflammation induced by LPS; the enhancement was greater with smaller nanoparticles	[109]
*In vivo*	TiO_2_ nanoparticles, gold nanoparticles	TDI-induced BALB/c asthma mice model	Intratracheal instillation at dose of 0.8 mg/kg once	TiO_2_ and Au nanoparticles increased pulmonary inflammation and airway hyperreactivity	[116]
